# Associated Corrective Procedures of Extra-Articular Asymptomatic Foot Malalignments in Total Ankle Replacement: Are They Really Mandatory?

**DOI:** 10.3390/jcm11154544

**Published:** 2022-08-04

**Authors:** Silvio Caravelli, Marco Di Ponte, Alberto Grassi, Stefano Zaffagnini, Massimiliano Mosca

**Affiliations:** II Clinic of Orthopaedics and Traumatology, IRCCS Istituto Ortopedico Rizzoli, 40136 Bologna, Italy

## 1. Introduction

In recent years, total ankle replacement (TAR) has undergone a series of evolutions and changes in terms of materials, prosthetic designs, and surgical techniques.

To improve implant survival and reduce the risk of revision, care should be taken in pre-operative planning of associated extra-articular deformities. Foot malalignments, including flat or cavus-varus foot, are frequent. When mild and completely asymptomatic, they raise the question: do or do they not need to be corrected as a protection function for the prosthetic components?

## 2. Discussion

Additional surgical procedures are frequently associated with TAR, and they include, among others, realignment osteotomies, tendon transfers, joint arthrodesis, and ligamentous reconstructions. “To perform or not to perform them (even when the patient is asymptomatic), that is the question”.

Satisfactory results in the TAR are obtained from the combination of several factors, including patient selection, prosthetic design, adequate rehabilitation, joint alignment, and ligamentous stability [1]. The last two are the only factors that strictly depend on the intraoperative management of the referring surgeon. While in the case of symptomatic extra-articular foot deformities, additional procedures are almost always necessary, to the detriment of residual post-operative pain, their actual need for asymptomatic extra-articular malalignments still raises doubts.

The still high rate of complications at long-term follow-up, compared to hip and knee prosthetic surgery, may depend, among other factors, on the imbalance of the definitive prosthetic ankle components [2,3]. Procedures such as calcaneal osteotomies, lateral and/or medial ligamentous reconstruction, and subtalar or midtarsal arthrodesis may be necessary even when the pathology is not symptomatic to protect the prosthetic components and reduce the risk of revision. In the most recent literature, the general failure rate is on average 12.3% at 7 years and 12.1% at 10 years [4,5]. However, the risk–benefit ratio connected to additional surgical procedures must be considered, according to a kind of “balance effect” that can move its needle towards the necessity of surgery or not, on the basis of a careful pre-operative and intra-operative assessment. The reasons against the execution of associated surgical procedures in asymptomatic foot malalignments include increased intraoperative risk due to the complexity of the surgery, increased soft tissue stress (with potential infectious or vascular complications) and operating time, and delayed bone consolidation or nonunion that could lead to revision bone surgery [6]. On the other hand, the elements in favor of additional surgery during TAR are represented by a better protection of the prosthetic components in the long term, thanks to a better distribution of load vectors and forces on the implant. In case of instability or extra-articular malalignments, they could lead to the failure of the implant, with periprosthetic osteolysis (generally due to repeated micromovements) and wear of the ultra-high-molecular-weight polyethylene (UHMWPE) liner [7,8,9].

Going into the details of the different prosthetic designs, a fixed-bearing prosthesis could worsen an asymptomatic osteoarthritis of the nearby joint and seems to be related to an increased risk of loosening, if compared to mobile-bearing design. However, in a recent meta-analysis, there were no statistically significant differences in the revision rate of the two different prosthetic designs [2,4,10]. Proper alignment and stability of the ankle are crucial for the protection of the prosthetic elements [1]. In case of failure of the prosthesis, revisioning the implant and not taking into consideration foot deformity or ligamentous laxity could lead to a new failure and poor clinical and radiographic results. [11,12].

In summation, the stakes are high. Revision TAR involves a more complex and risky surgery, since the bone stock is limited [6] and the lower limb symmetry is compromised, requiring experienced surgeons to manage it. The aim of this short editorial is to raise points of view, discussions, and questions, with the need for further in-depth and long-term studies regarding the use of associated procedures in case of asymptomatic foot deformities, instability, or osteoarthritis to reduce the incidence of revision surgery in the long-term follow-up.
